# Corroboration of the Toms effect from a frictional drag reducing self-polishing copolymer

**DOI:** 10.1038/s41598-023-36549-8

**Published:** 2023-06-07

**Authors:** Hyun Park, Donghyun Cho, Jong-Woon Ha, Do-Hoon Hwang, Ra Hui Park, Hwawon Seo, Inwon Lee

**Affiliations:** 1 Department Naval Architecture and Ocean Eng, Pusan National University, Busan, 46241 Korea; 2 Korea Research Institute of Chemical Technology, Daejon, 34114 Korea; 3 Department Chemistry, Pusan National University, Busan, 46241 Korea; 4 Material and Coating Research, Samsung Heavy Industries Co. Ltd., Gyeongsangnam-Do, 53261 Korea

**Keywords:** Mechanical engineering, Sustainability

## Abstract

A novel frictional drag reducing self-polishing copolymer (FDR-SPC) was first developed by the authors. The FDR-SPC is a special derivative of an SPC that was designed to achieve skin frictional drag reduction in turbulent water flow by releasing polyethylene glycol (PEG) into water through a hydrolysis reaction. Thus, the FDR-SPC coating acts as a continuous medium accommodating countless, molecular-level polymer injectors. However, direct evidence of such PEG release has not yet been demonstrated. Here, we report the results of in situ PEG concentration measurement based on the planar laser-induced fluorescence (PLIF) method. Polyethylene glycol methacrylate (PEGMA) was probed by the fluorescent functional material dansyl, and the fluorescence intensity from dansyl-PEG was then measured to quantify the concentration in the flow. The near-wall concentration of dansyl-PEG is observed to range from 1 to 2 ppm depending on the flow speed, which corroborates the existence of a drag reducing function for the FDR-SPC. In the concurrent measurement of skin friction, the present FDR-SPC specimen exhibited a skin friction reduction ratio of 9.49% at the freestream flow speed $U=5m/s$. In the comparative experiment of dansyl-PEGMA solution injection, the skin friction was found to decrease by 11.9%, which is in reasonable accordance with that for the FDR-SPC.

## Introduction

Frictional drag reduction is of utmost importance for ships in terms of energy economy and greenhouse gas emissions. The largest energy demand on ships is to overcome drag during navigation, which consists of frictional, pressure and wavemaking components. The contribution from the frictional drag is the largest, approximately 80% for modern hull forms. Based on international marine bunker statistics^1^, Park and Lee^2^ estimated that a 10% reduction in frictional drag for the entire world fleet would result in fuel savings worth 4.7 billion US$/year. The dominance of skin frictional drag is due to the energetic momentum transfer in high Reynolds number turbulent flow. Among the various control strategies for attenuation of turbulent flow activity, henceforth reduction of skin frictional drag, proposed over several decades, polymer injection is regarded as the most successful. The technique was first introduced by Toms^3^, who found that the addition of a few ppm of a high-molecular-weight polymer to a turbulent water flow can lead to a significant reduction in skin friction by as much as several tens of percent. Long chain polymer molecules dissolved in water extract energy from the turbulent near-wall flow via coiling of their molecular structures and then release energy via stretching back into the flow. Various studies have investigated the drag reduction efficiency of polymer injection in turbulent flows^4–7^. Notably, not only conventional bulk flow dilution but also near-wall injection is effective in reducing skin friction. However, the injected drag reducing agent is prone to diffuse out of the near-wall region, which leads to degradation of the skin friction reduction effect with increasing downstream distance.

There have been notable researches to achieve skin friction reduction by means of coating. The coating can be functioning as continuous medium to inject drag reducing substance, thereby avoiding the degradation effect of the localized injection method. The first example was a PEO (polyethylene oxide) powder mixed coating devised by Yang et al.^8^, which led to a skin friction reduction over 10%. In their coating, however, the PEO powders were physically mixed with the coating matrix, thereby giving rise to an increase in surface roughness and rapid release associated with the high solubility of PEO in water. Consequently, the skin friction reduction almost disappeared after a few days. Recently, Rowin et al.^9^ proposed another novel idea in that drag-reducing polymer chains were bonded on a metallic surface, leading to a 19% drag reduction in turbulent channel flow. However, it was reported that the drag reduction gradually diminished within an hour.

The frictional drag reducing self-polishing copolymer (FDR-SPC), which was first synthesized by the authors^10,11^, is a revolutionary alternative in which the injection mechanism is replaced by a novel chemical release mechanism. The SPC, which is the base polymer of the FDR-SPC, forms a gradually dissolvable layer through a hydrolysis reaction with water. With the progress of the hydrolysis reaction, the outermost layer of the SPC resin becomes hydrophilic and subsequently delaminates, exposing the fresh surface underneath. These sequential actions comprise an erosion process, which is the most distinctive feature of the SPC resin to ensure stable release of its constituents, such as toxic antifoulants, over such a prolonged period of time as a few years. Owing to the erosion process, the SPC has been extensively used for the matrix of marine antifouling coatings. The FDR-SPC was synthesized by copolymerizing the FDR functional group of polyethylene glycol methacrylate (PEGMA) with other SPC monomers, such as methyl methacrylate (MMA) and silyl methacrylate (SMA). PEGMA was adopted as a FDR functional group for two reasons. First, it contains the same polyethylene chain molecular structure as polyethylene oxide (PEO), which has been widely employed for polymer injection^4–7^. According to Kenis and Hoyt^4^, PEO is noted to cause a significant drag reduction of tens of percent even at a very dilute concentration of a few ppm. Second, PEGMA contains methacrylate (MA), whose double bond could be used for copolymerization.

**Figure 1:**
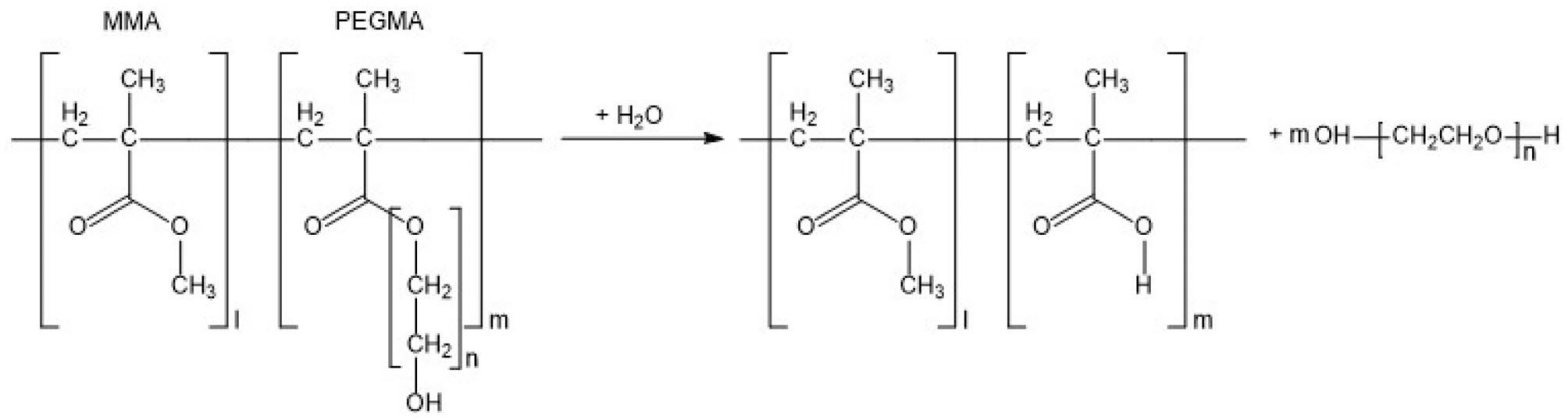
Hydrolysis reaction of the FDR-SPC.

Figure 1 presents the reaction mechanism underlying PEG release from the FDR-SPC. Being chemically bonded to the FDR-SPC, PEG is not released into the water until the hydrolysis reaction breaks the chemical bond of PEGMA. Therefore, PEG release into water flow is governed by the abovementioned erosion process, thereby ensuring persistent skin friction reduction as long as the coating film exists. In summary, the present FDR-SPC is the embodiment of the molecular injection mechanism for FDR agents. Lee et al*.*^10^ reported that the FDR-SPC exhibits skin friction reduction as large as 13.5% over smooth surfaces. Furthermore, a significant reduction in the turbulence intensity was demonstrated for the FDR-SPC, corroborating the presence of the Toms effect caused by the FDR-SPC. Based on this FDR-SPC, an FDR antifouling (FDR-AF) marine coating was subsequently developed and evaluated in terms of the full-scale energy saving performance during service of a 176 k DWT bulk carrier^12^. To quantify the effect of the FDR-AF coating on the ship speed performance, Cho et al.^12^ carried out ISO19030 standard analysis for the navigation data collected from the vessel over five years (November 2014 through December 2019). Notably, the authors were able to isolate the effect of the FDR-AF coating by comparing two identical freshly coated vessels immediately after redocking with only the coatings being changed. The coating leads to a speed increase of 3.72% over the conventional AF coating, which is equivalent to a power (fuel) savings of 11.7%.

Although the hydrodynamic performance of the FDR-SPC has been confirmed in various environments encompassing laboratory tests^10^ and in-service full-scale vessel application^12^, the underlying physical mechanism of PEG release has not been identified. This is due to the lack of in situ measurement capability for the chemical concentration in the presence of water flow. We propose a novel planar laser-induced fluorescence (PLIF) method in which the concentration of the released PEG can be quantified in terms of the intensity of the fluorescence emitted thereof.

## Materials and methods

### Labelling of PEGMA with the dansyl group

Toward the quantitative in-situ concentration measurement of PEGMA from FDR-SPC, the PEGMA for FDR-SPC synthesis was labelled with a dansyl fluorophore. All the starting materials and reagents were purchased from commercial sources, such as Alfa Aesar, TCI, and Sigma Aldrich. ^1^H nuclear magnetic resonance (NMR) spectra were measured on a Varian Mercury Plus 300 MHz spectrometer. The UV–Vis absorption spectrum was obtained on a JP/UV-1800 UV/Vis spectrometer analyser. The photoluminescence (PL) spectrum was obtained on an F-7000 FL spectrophotometer.

Dansyl chloride (8.0 g, 29.6 mmol, 1.0 eq) and PEGMA (MW ≈ 360 g/mol, 10.7 g, 1.0 eq) were dissolved in dichloromethane (DCM) (300 mL). To the mixture, 1,4-diazabicyclo[2,2,2]octane (DABCO) (2.5 g, 22.2 mmol, 1.2 eq) was added, and the mixture was stirred at 25 °C for 4 h. The mixture was extracted with DCM and dried over MgSO_4_. After removal of the solvent, the crude product was purified by column chromatography over silica gel (eluent: DCM only and methanol only) to give a pure product as yellow oil (15.5 g, 88%). ^1^H NMR (300 MHz, CDCl_3_, ppm): *δ* 8.61 (d, 1H, *J* = 9.0 Hz), 8.28 (d, 2H, *J* = 8.7 Hz), 7.61 (m, 2H), 7.22 (d, 1H, *J* = 7.2 Hz), 6.13 (s, 1H), 5.58 (s, 1H), 4.31 (t, 2H, *J* = 5.4 Hz), 4.11 (t, 2H, *J* = 4.5 Hz), 3.61 (m, 32H), 2.89 (s, 6H), 1.95 (s, 3H). ^13^C NMR (75 MHz, CDCl_3_, ppm): *δ* 167.30,151.71, 136.09, 131.54, 131.19, 130.44, 130.42, 129.75, 128.61, 125.75, 123.01, 119.41, 115.49, 77.60, 72.33, 70.46, 69.51, 69.05, 68.49, 68.28, 67.97, 63.85, 61.63, 53.56, 45.37, 18.29.

**Figure 2:**
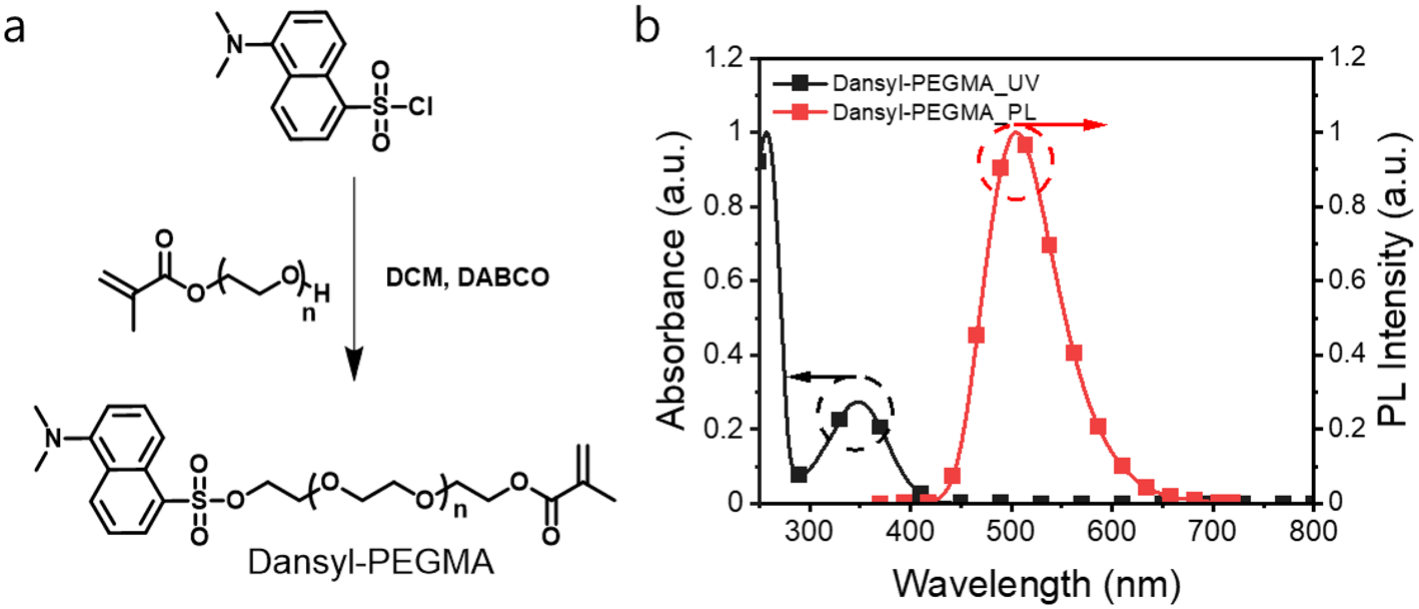
Labelling of PEGMA with the dansyl group. (**a**) Synthetic route and chemical structure of dansyl-PEGMA. (**b**) Absorption and emission spectra of dansyl-PEGMA.

UV–Vis absorption and PL spectra of dansyl-PEGMA were measured in the solution state (in chloroform). The peak wavelengths of the absorption and emission spectra of dansyl-PEGMA were determined to be 347 nm and 503 nm, respectively (see Fig. 2).

### Concurrent measurements of concentration and skin friction in the high-speed water tunnel

**Figure 3:**
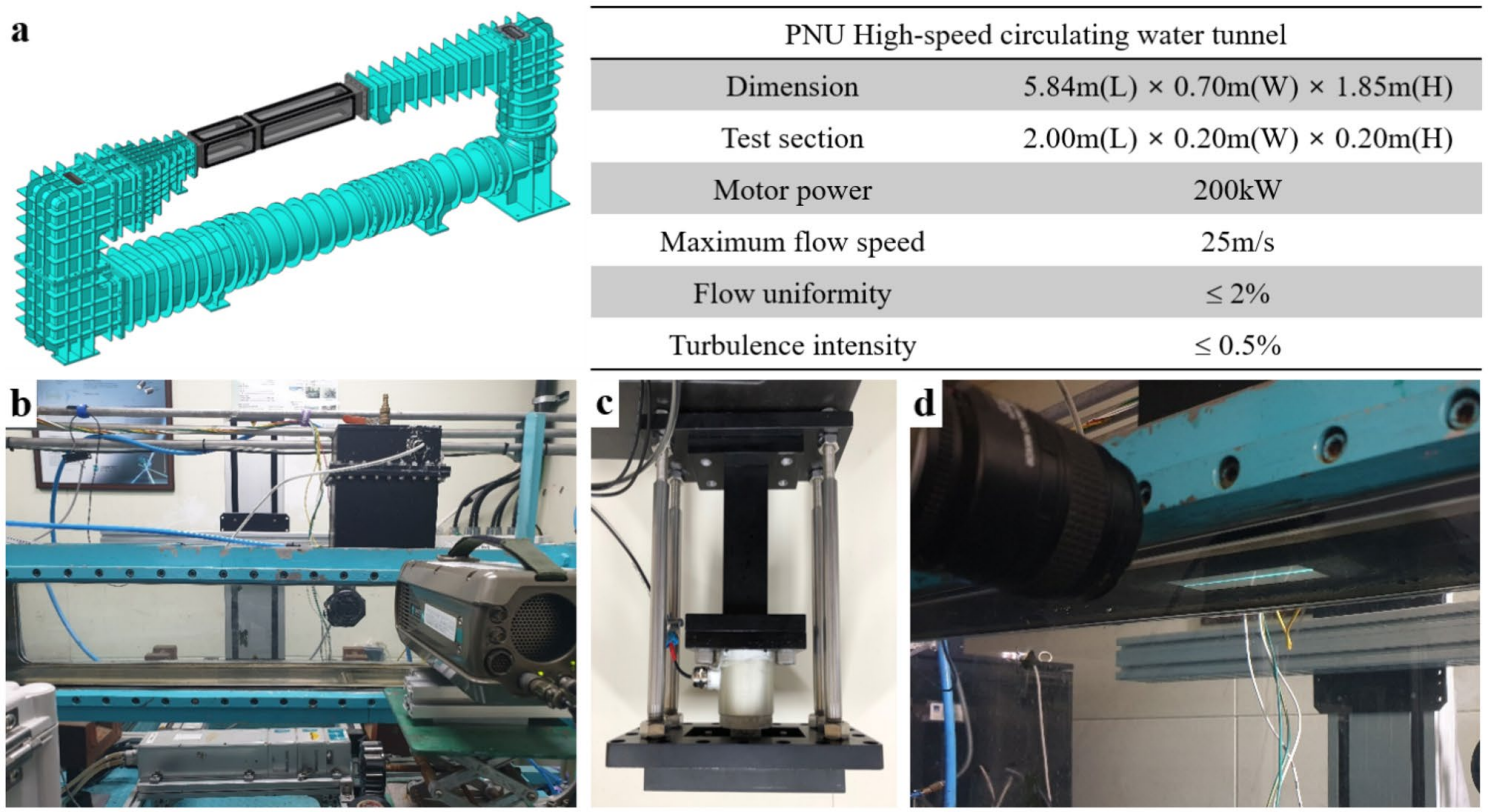
High-speed water tunnel of Pusan National University. (**a**) 3-D model and specifications. (**b**) Photo of the test section. (**c**) Floating-element balance for skin friction measurement. (**d**) Photo of the PLIF measurement for an FDR-SPC sample. The background illumination is given only in reference to the overall configuration.

The PRD3-S9D-coated plate was installed in the test section of the high-speed water tunnel of Pusan National University (see Fig. 3). Being mounted underneath the floating-element balance (Fig. 3c), the coated plate formed a flush-mounted floating element parallel to the top wall of the test section. Contact with water at the surface of the PRD3-S9D coating initiates the hydrolysis reaction dissociating dansyl-PEGMA into dansyl-PEG, which is subsequently released into water flow. Upon being irradiated by a UV laser light sheet, the released dansyl-PEG emits fluorescence light whose intensity is proportional to the concentration in water flow. Figure 3d portrays such a measurement, except that the background illumination is not given in the actual measurement. To convert the fluorescence intensity to the dansyl-PEG concentration, a calibration test should be performed prior to the PLIF measurement.

The PLIF system used a 10-bit intensified CMOS camera with a resolution of 1280 × 1024 pixels, a dual-head pulsed Nd:YAG laser operating at 355 nm and suitable laser light sheet generation optics. The released dansyl-PEG (FDR-SPC case) or dansyl-PEGMA (mechanical injection case) fluoresces under the action of the irradiating 355 nm laser light sheet. The emitted light of 503 nm wavelength was captured using the CMOS camera. The field of view of PLIF image corresponded to 25 mm × 20 mm in the streamwise and wall-normal directions, respectively.

**Figure 4:**
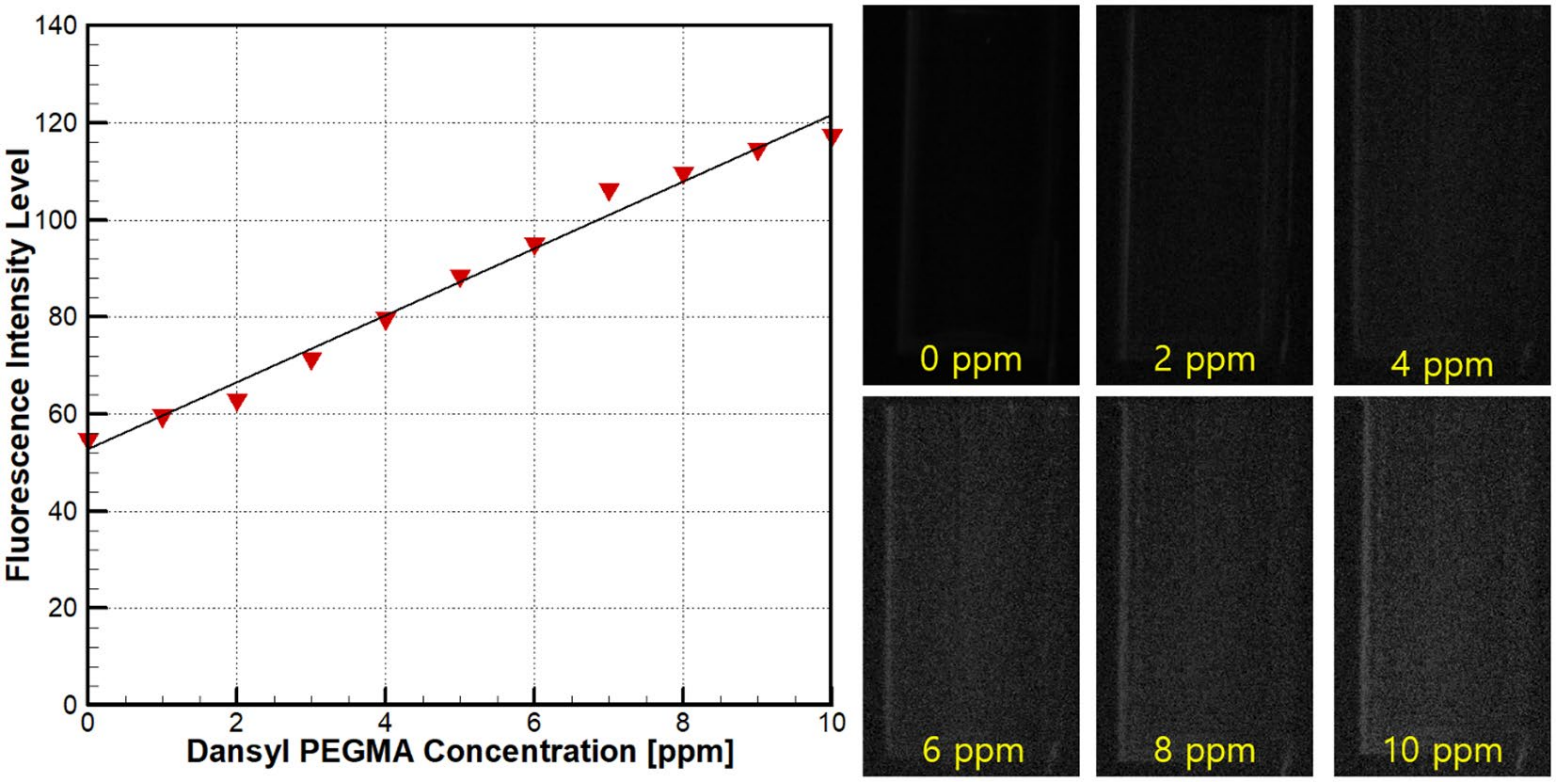
Calibration curve for the PLIF measurement and corresponding standard samples.

Prior to the measurement, the present PLIF system was calibrated using standard PEGMA solution with known concentration; crystal vials containing PEGMA solution with concentration varying from 0 to 10 ppm were situated at the measurement location in the test section. The acquired fluorescent images were processed with respect to the intensity level. Figure 4 presents a typical calibration curve between the fluorescent intensity and PEGMA concentration, together with corresponding fluorescent images of the standard samples.

In parallel with the PLIF measurement, the skin frictional drag force ${F}_{F}$ acting on the surface was obtained by measuring the side force on the plate using the balance. The average skin frictional stress ${\tau }_{w}$ was then obtained by dividing the skin frictional drag force by the area of the plate. The disturbance of the side force reading due to the gap and height difference between the specimen and the adjacent wall must be minimized. Great care was exercised in the installation of the coated plate to keep the gap and the height difference below 10 μm and 5 μm, respectively. Profiles of the streamwise mean velocity and fluctuations were also measured using laser Doppler velocimeter (LDV), as shown in Fig. 3b.

### Rotor test for erosion rate measurement

**Figure 5:**
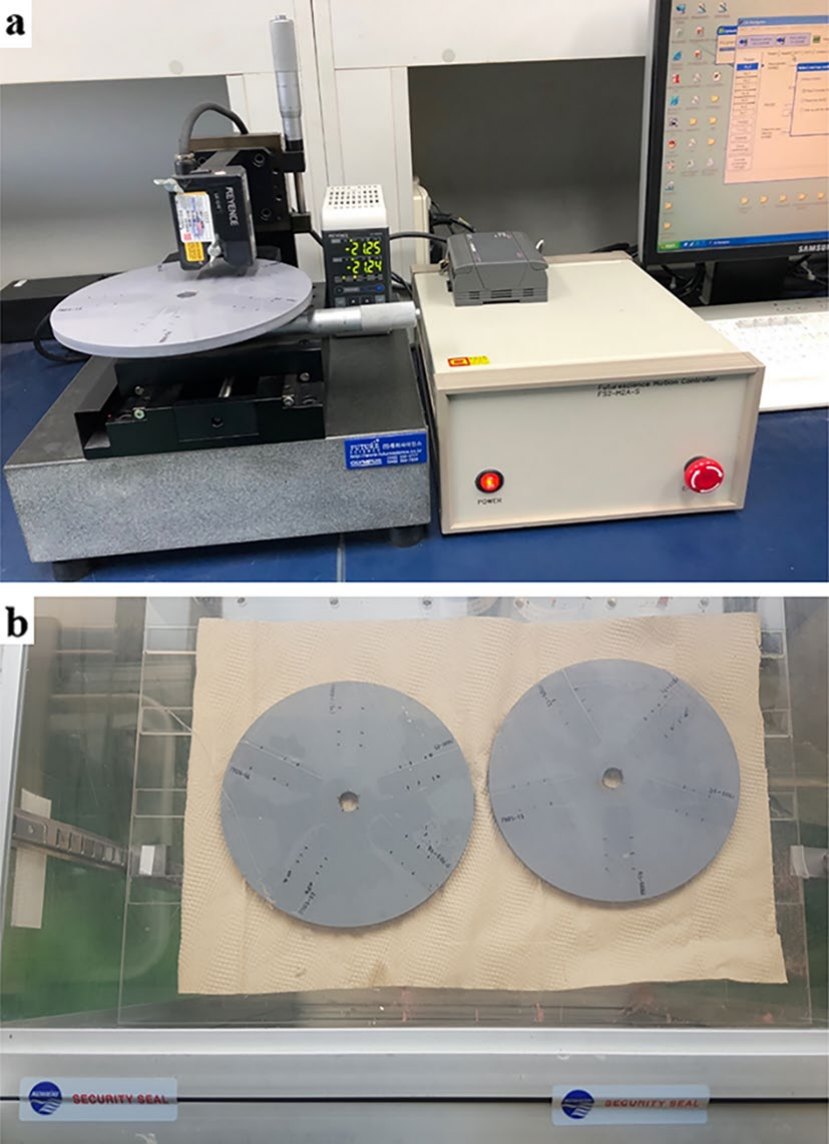
Procedures of the rotor test. (**a**) Coating thickness measurement using a laser CCD displacement sensor. (**b**) Desiccation of a rotor specimen under KOMERI supervision.

To evaluate the erosion performance of the synthesized resin, the experiment was conducted as per the procedure outlined in the ASTM D4939 standard (Standard test method for subjecting marine antifouling coating to biofouling and fluid shear forces in natural seawater). A 300 $\mu m$ aliquot of resin was applied by the applicator on the disc and dried for one week at room temperature. The thickness of the resin was measured using a laser CCD displacement sensor (LK-G15, KEYENCE; Fig. 5a). The disc was connected to the motor using a shaft and rotated nonstop at 300 rpm in artificial sea water (ASW) for 2 weeks. Subsequently, the disc was washed several times with DI water to wash away any adhering salts and subsequently dried for 72 h under ambient conditions (Fig. 5b), and the thickness of the resin was remeasured. This was repeated for four cycles. To guarantee compliance with the ASTM D4939 standard, the entire process of the test was supervised by experts from the Korea Marine Equipment Research Institute (KOMERI), which is a KOLAS (Korea Laboratory Accreditation Scheme) certification organization. The test results were compiled and reported in the Test Certificate of KOMERI-0311-18T4564.

### Comparative experiment of dansyl-PEGMA solution injection

**Figure 6:**
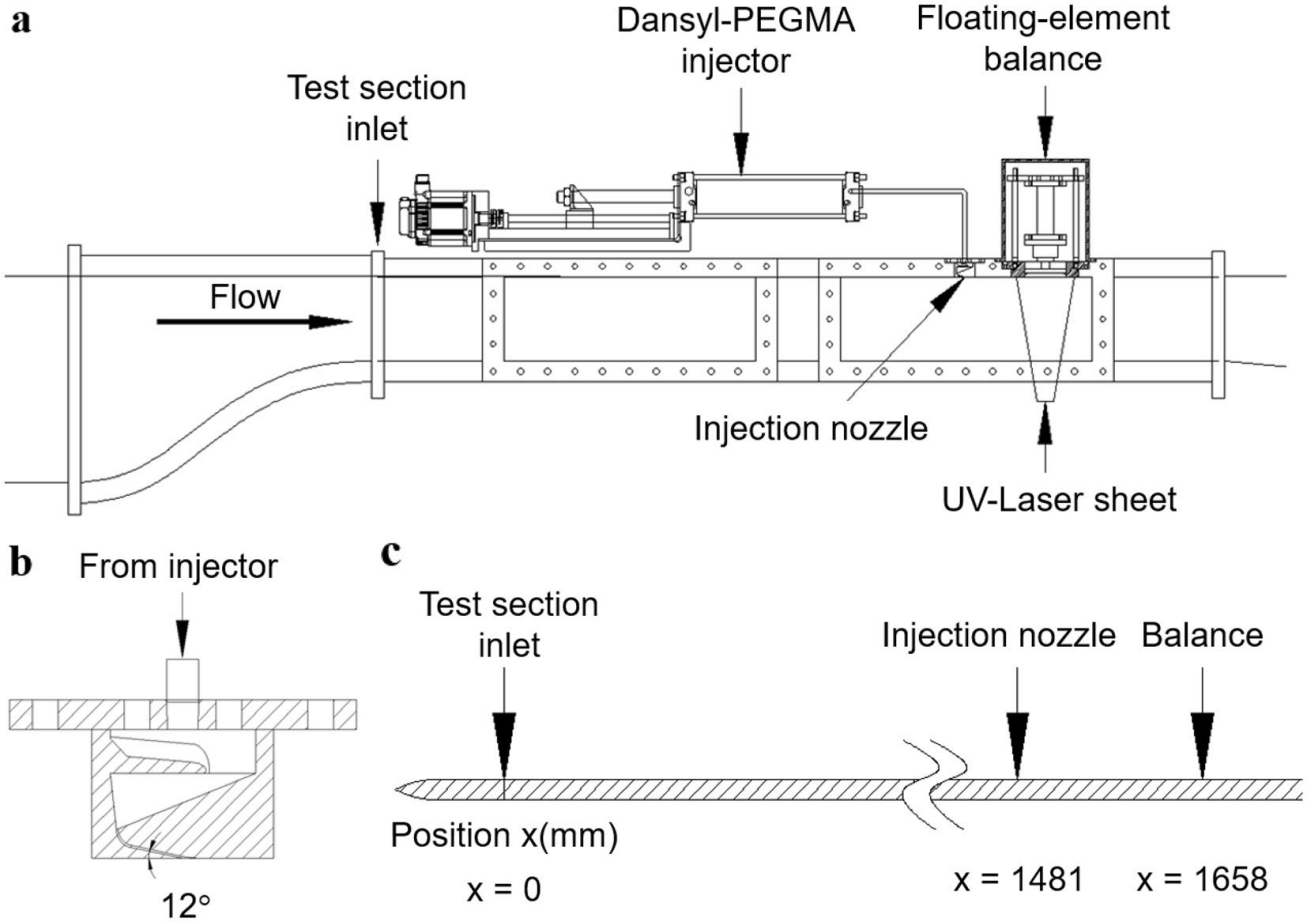
Schematic diagram of the PEGMA injection test apparatus. (**a**) Entire setup. (**b**) Details of the injection slot. (**c**) Schematic showing the test section wall as a boundary layer flat plate, the location of the injection nozzle and the measurement location.

In this experiment, the dansyl-PEGMA solution was directly injected through a rectangular slot upstream of the floating-element balance. Thus, the so-called molecular-level PEG injection mechanism of the FDR-SPC was substituted with the mechanical injection system. The injection slot, which was 0.5 mm wide and 50 mm long, was placed 177 mm upstream of the location for skin friction and PLIF measurements (Fig. 6). The conditions for dansyl-PEGMA solution injection were chosen with reference to those in the PEO solution injection study of Sommandepalli^7,13^. For instance, the injection volume flow rate ${Q}_{i}$ was set at three values of ${Q}_{i}=0.60{Q}_{S}, 0.75{Q}_{S}$ and $0.90{Q}_{S}$, where ${Q}_{S}=67.3\nu$ is the volume flow rate in the viscous sublayer up to ${y}^{+}=11$. This was aimed at minimizing undesirable disturbances to the boundary layer flow due to the injected flow velocity. The freestream flow speed was fixed at $U=5m/s$. In addition, the concentration of dansyl-PEGMA solution ${\varphi }_{i}$ was chosen to be 3.4 ppm, which is approximately twice the concentration measured for $U=5m/s$. This was to compensate for the downstream concentration decrease due to cross-stream diffusion in the mechanical injection.

## Results and discussion

### Measurement of the concentration of drag reducing agent released from the FDR-SPC

Toward the quantitative in-situ concentration measurement of PEGMA from FDR-SPC, the PEGMA for FDR-SPC synthesis was labelled with a dansyl fluorophore, as described in Section “Labelling of PEGMA with the dansyl group”. The resulting dansyl-labelled FDR-SPC, hereinafter termed PRD3-S9D, was coated onto smooth aluminium plates 100 mm long, 50 mm wide and 5 mm thick. The PRD3-S9D-coated plate was installed in turbulent boundary layer flow, and in situ measurements of the concentration in the turbulent boundary layer flow were performed in a rectangular area of 25 mm × 20 mm. The wall-normal measurement region extended to the outer region of the turbulent boundary layer, ranging from roughly $0.62\delta$ (for $U=2$ m/s) to $0.82\delta$ (for $U=7$ m/s).

**Figure 7:**
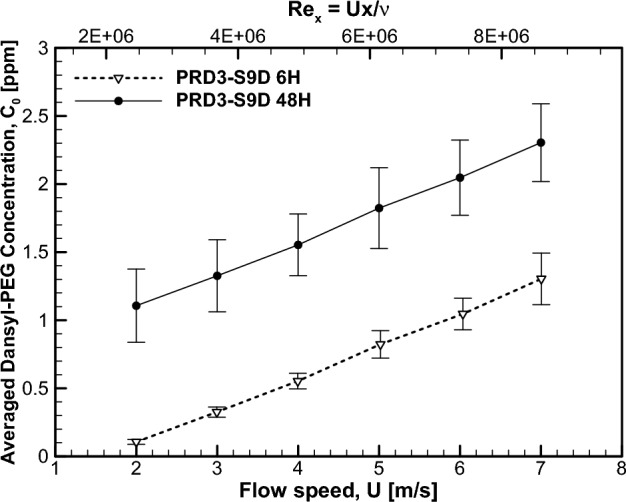
Average concentration of dansyl-PEG for the PRD3-S9D specimen.

Figure 7 presents such results. Notably, the initial concentration of dansyl-PEG inside the test section was zero. With the progress of the hydrolysis reaction, the concentration increased until a certain equilibrium level was reached. To identify the time-dependent release and diffusion mechanism of dansyl-PEG, we varied the immersion time of the PRD3-S9D coating prior to the PLIF measurements among three levels: 6, 48 and 96 h. As seen in Fig. 7, two sets of concentration data with immersion times of 6 h (denoted as ‘PRD3-S9D 6H’) and 48 h (‘PRD3-S9D 48H’) are plotted with respect to the freestream flow speed $U$ and the streamwise Reynolds number ${Re}_{x}=\frac{Ux }{\nu }$. Here, $x$ is the streamwise distance between the leading edge of the test section and the measurement location, and $\nu$ is the kinematic viscosity of water. In this study, uncertainty analysis of the PLIF result was performed considering the random error in the acquired fluorescence images combined with the bias limits estimated using the methods of Vaidyanatham et al.^14^. A closer inspection of Fig. 7 reveals that as the immersion time increases from 6 to 48 h, the concentration increases by approximately 1 ppm regardless of the flow speed. The concentration data after 96 h of immersion were found to be almost identical to those at 48 h, so they are not plotted in Fig. 7 for the sake of brevity. These results suggest that PEG release becomes fully activated after 48 h of immersion.

The dansyl-PEG concentration is observed to be almost linearly proportional to the flow speed for both sets of data. This is attributable to the dependence of the erosion process on the flow speed. The erosion rate is defined as the speed at which the coating thickness decreases over time due to continued mass release and is usually measured by means of a rotor test based on the ASTM D4939 standard (see Section “Rotor test for erosion rate measurement” for experimental details). The erosion rate of the present FDR-SPC at a rotor speed of 5 m/s was found to be 4.84 μm/month. Since PEG release is the consequence of the hydrolysis reaction, which comprises the erosion process, its flux can be estimated from the erosion rate. Combining the erosion rate with the density of PRD3-S9D leads to the mass of PRD3-S9D released per unit area and time, which amounts to $0.0497g/\left(c{m}^{2}\cdot month\right)$. This can be further converted into the PEG mass flux ${J}_{0}=0.767\times {10}^{-7} g/\left({m}^{2}\cdot sec\right)$ by considering the molecular weight of dansyl-PEGMA. Kiil et al.^15^ demonstrated that the erosion rate of the SPC is generally proportional to the flow speed. Consequently, the PEG mass flux ${J}_{0}$ and the PEG concentration in the flow will become proportional to the flow speed, which is the case in Fig. 7.

The linear dependence between the concentration and the flow speed is of profound significance in that the present chemical PEG release system adjusts itself to the incoming freestream flow speed. For mechanical polymer injection through a 2-dimensional slot, Somandepalli et al.^7^ noted that the drag reduction effect is governed by the nondimensional parameter $K=\frac{Q{C}_{m} }{\rho UX}$. Here, $Q$ is the volume flux of injected polymer solution per unit span, ${C}_{m}$ is the mass concentration of the polymer solution, and $X$ is the distance between the measurement location and the injection slot. According to this model, ${C}_{m}$ should be linearly proportional to the flow velocity $U$ to maintain the drag reduction effect regardless of $U$. This is exactly what happens in the present polymer release system using the FDR-SPC. The aforementioned degradation of the skin friction reduction effect with increasing downstream distance is represented by the relation $K\sim 1/X$. The present case of the FDR-SPC, however, is not prone to such a distance effect because the near-wall flow region is ‘locally’ fed with PEG solution by the continuous FDR-SPC coating underneath the flow.

**Figure 8:**
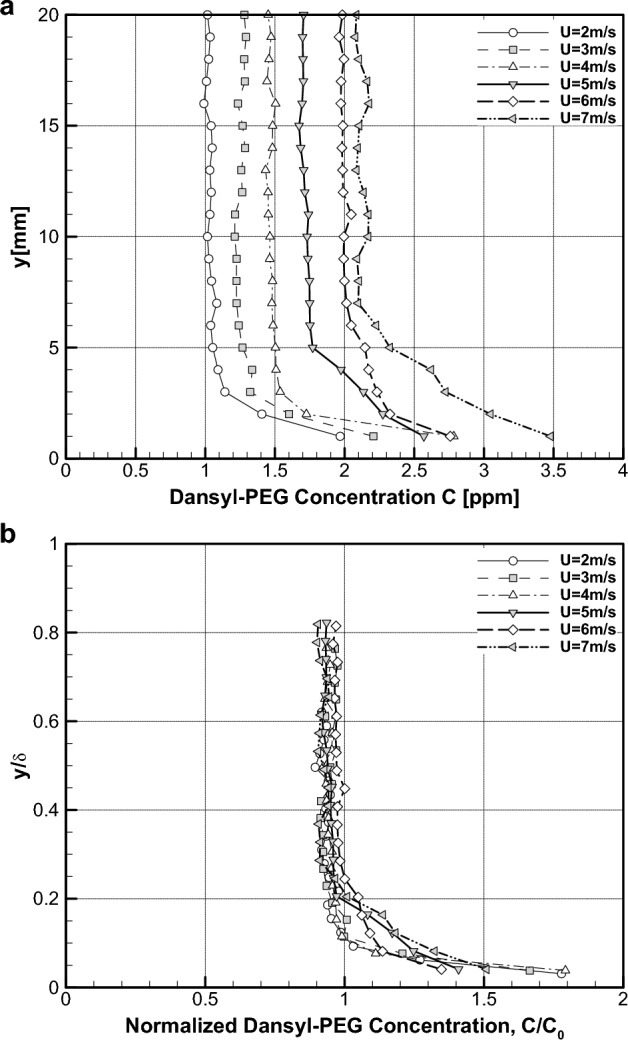
Concentration distribution of dansyl-PEG. (**a**) Dimensional distribution. (**b**) Nondimensional distribution.

The PLIF images were further subdivided in the wall-normal direction and then processed to get the wall-normal concentration profile shown in Fig. 8. The dimensional profile is presented in Fig. 8a and its nondimensional version $C/{C}_{0}$ vs. $y/\delta$ being plotted in Fig. 8b. Here, ${C}_{0}$ means the average concentration given in Fig. 7. In Fig. 8a, all concentration profiles are noted with a large near-wall gradient and convergence to constant values in the outermost region. In addition, the local concentrations are roughly proportional to flow velocity, which is similar to that of average concentration in Fig. 7. These behaviours suggest the self-similarity of local concentration distribution, which is substantiated in the normalized plot of Fig. 8b. The self-similar concentration distribution of dansyl-PEG is another evidence of wall-normal mass flux of PEG in the turbulent boundary layer flow. Also, this is in support of the above-mentioned sustainability of skin-friction reduction in higher Reynolds number.

**Figure 9:**
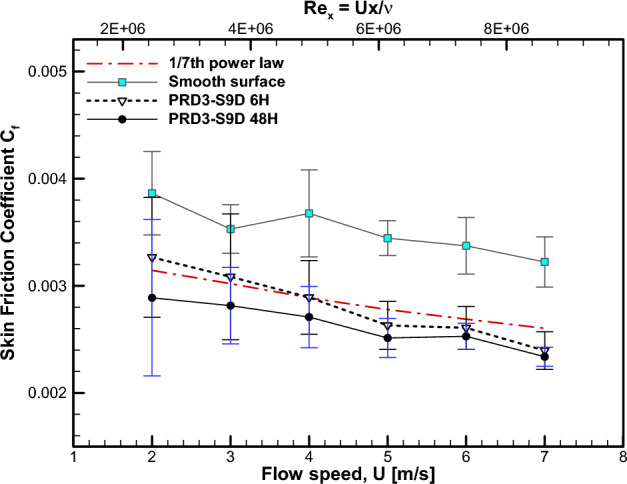
Skin friction coefficient ${C}_{f}=\frac{{\tau }_{w} }{0.5\rho {U}^{2}}$ for the PRD3-S9D specimen in comparison with experimental results and 1/7th power law correlation for smooth surface.

The results of concurrent measurement of skin friction for the PRD3-S9D specimen with two immersion times are plotted in comparison with the baseline measurement of smooth specimen in Fig. 9. Here, the nondimensional skin friction coefficient ${C}_{f}=\frac{{\tau }_{w} }{0.5\rho {U}^{2}}$ is plotted against both the Reynolds number ${Re}_{x}$ and the freestream flow speed $U$. It is obvious that the two data sets from the PRD3-S9D specimen exhibits significant skin friction reduction compared with the smooth surface. Figure 9 also presents the empirical correlation of the 1/7th power law ${C}_{f,ref}=\frac{0.027 }{{Re}_{x}^{1/7}}$, which is referenced to the baseline case of a smooth surface^16^. Again, the two sets of data exhibit values of ${C}_{f}$ smaller than ${C}_{f,ref}$, which supports the skin friction reduction due to the FDR-SPC. In addition, the 48-h immersion data exhibit smaller skin friction than the 6-h immersion data. In connection with the increase in the PEG concentration with increasing immersion time, this suggests that the amount of skin friction reduction is correlated with the PEG concentration in water, thereby corroborating the Toms effect by the present FDR-SPC. The percentage of skin friction reduction, which is defined as $\left(1-{C}_{f}/{C}_{f,ref}\right)\times 100$ (%), is enumerated in Table 1.

**Table 1:** Percentage of skin friction reduction for the PRD3-S9D specimen.

Flow speed $U$ (m/s)	${Re}_{x}$	Skin friction reduction $\left(1-{C}_{f}/{C}_{f,ref}\right)\times 100$ (%)	
		PRD3-S9D 6H	PRD3-S9D 48H
2.0	2.456 × 10^6^	− 3.87	8.15
3.0	3.769 × 10^6^	− 2.10	6.81
4.0	4.907 × 10^6^	− 0.13	6.24
5.0	6.141 × 10^6^	5.27	9.49
6.0	7.382 × 10^6^	2.90	5.83
7.0	8.602 × 10^6^	7.96	10.17

A relatively large levels of uncertainty in ${C}_{f}$, typically as large as a few tens of percent, were obtained for the two PRD3-S9D cases shown in Fig. 9. This is in contrast to the smaller uncertainty for the baseline case shown in Fig. 9, which is approximately several percent. This discrepancy is attributable to the differences in the measurement duration for the baseline case and PRD3-S9D cases. For the PRD3-S9D cases, the accumulation of PEG solution in the recirculating freestream due to the PEG previously released from the coating was anticipated. Being far from the normally experienced external ship flow conditions, which are devoid of freestream PEG concentration, this situation should be avoided. Consequently, the measurement duration for the FDR-SPC specimen was limited to several seconds to minimize the change in the flow conditions due to water recirculation. This led to a relatively small number of samples for the skin friction measurement of the FDR-SPC specimen. In contrast, the baseline case measurement was performed for a longer period to obtain a larger number of samples. The resulting difference in the number of samples led to different levels of measurement uncertainty.

**Figure 10:**
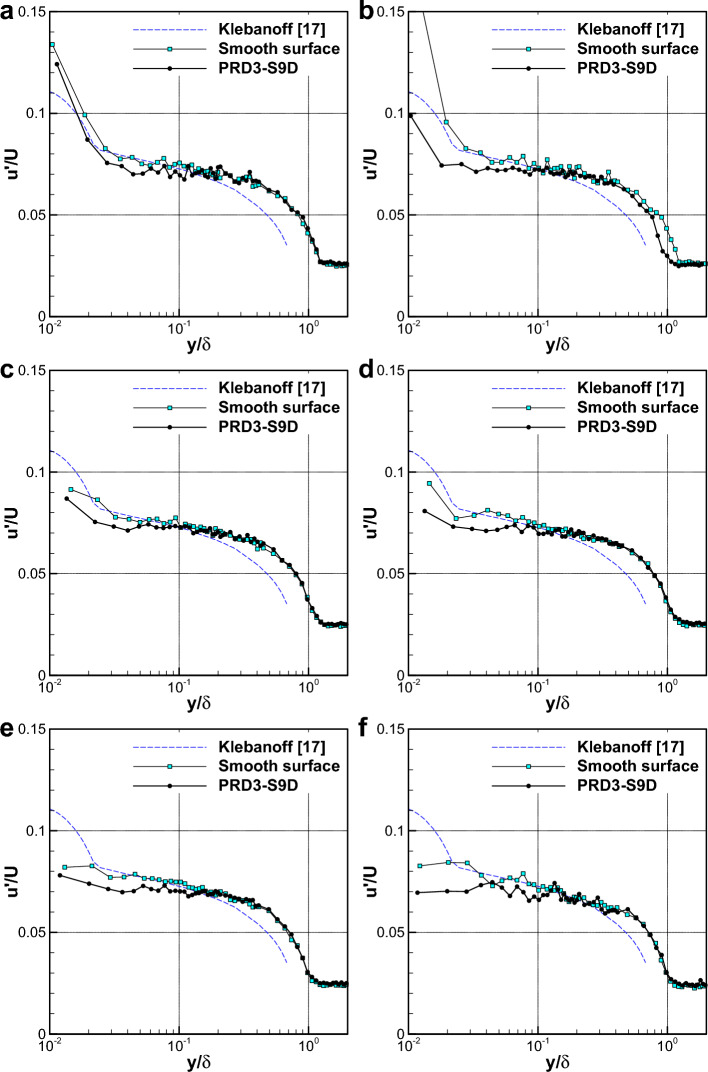
Comparison of the profiles of streamwise velocity fluctuations for smooth surface and PRD-S9D specimen. (**a**) $U=2\mathrm{ m}/\mathrm{s}$. (**b**) $U=3\mathrm{ m}/\mathrm{s}$. (**c**) $U=4\mathrm{ m}/\mathrm{s}$. (**d**) $U=5\mathrm{ m}/\mathrm{s}$. (**e**) $U=6\mathrm{ m}/\mathrm{s}$. (**f**) $U=7\mathrm{ m}/\mathrm{s}$.

Figure 10 presents the comparison between the profiles of normalized streamwise velocity fluctuations $\frac{\sqrt{\overline{{u }^{2}}} }{U}$ for the smooth surface and the PRD3-S9D. Here, the data from the zero-pressure gradient turbulent boundary layer in Klebanoff^17^ is also plotted for validation of the smooth surface data. It is observed that the FDR-SPC leads to reduced turbulent fluctuations particularly in the inner layer $\frac{y }{\delta }<0.1$. This is associated with the observed concentration of PEG in the turbulent boundary layer and supportive of the skin friction reduction effect shown in Fig. 9.

### Corroboration experiment of skin friction reduction by means of mechanical injection

To corroborate the skin friction reduction capability of dansyl-PEGMA, we carried out a comparative experiment (see Section “Comparative experiment of dansyl-PEGMA solution injection” for experimental details). The injection parameters are listed in Table 2. Here, the PEGMA injection mass flux ${J}_{i}$ is defined by multiplying ${\varphi }_{i}$ by the injection velocity ${V}_{i}={Q}_{i}/{A}_{i}$. This quantity is directly comparable to the PEG release mass flux ${J}_{0}$ for the FDR-SPC. Included in Table 2 is the water injection case with ${Q}_{i}/{Q}_{S}=0.75$ and ${\varphi }_{i}=0.0$, which was carried out to verify the effect on skin friction.

**Table 2:** Conditions and results for the dansyl-PEGMA solution injection test.

${J}_{i}$(g/m^2^⋅s)	${Q}_{i}/{Q}_{S}$	${V}_{i}$(m/s)	Injected concentration ${\varphi }_{i}$ (ppm)	Measured concentration $\varphi$ (ppm)	Skin friction reduction $\left(1-{C}_{f}/{C}_{f,0}\right)\times 100$ (%)
0.0	0.75	0.10	0.0	–	–
0.272 × 10^−6^	0.60	0.08	3.4	2.33	11.90
0.340 × 10^−6^	0.75	0.10	3.4	2.56	15.77
0.408 × 10^−6^	0.90	0.12	3.4	2.86	17.10

**Figure 11:**
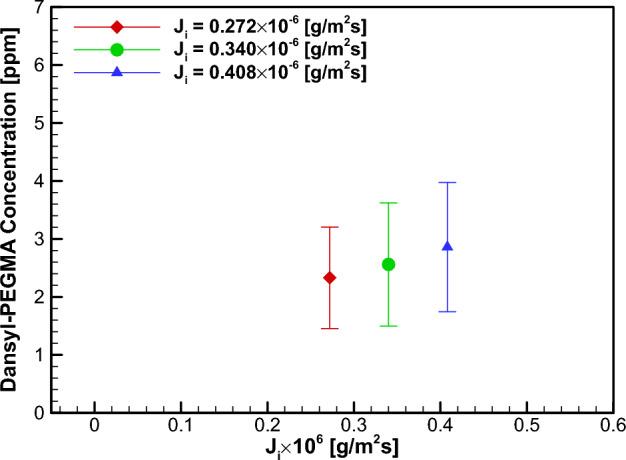
Measured concentration of dansyl-PEGMA for the PEGMA solution injection case.

**Figure 12:**
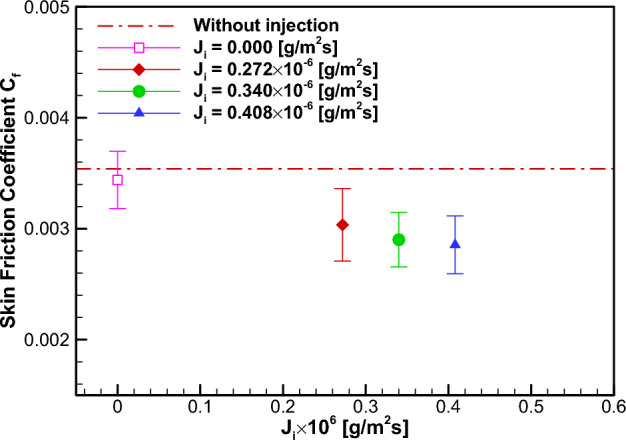
Skin friction coefficient ${C}_{f}=\frac{{\tau }_{w} }{0.5\rho {U}^{2}}$ for the PEGMA solution injection case.

Figure 11 and Table 2 present the results of PLIF concentration measurement for the injection test. The measured PEGMA concentration $\varphi$ in the flow was found to be smaller than the injected concentration ${\varphi }_{i}$. This is due to the cross-stream diffusion of PEGMA during convection downstream to the measurement position. Note that the case ${J}_{i}=0.272\times {10}^{-6}g/\left({m}^{2}\cdot sec\right)$ yielded a concentration value similar to that in the FDR-SPC case. The corresponding skin friction measurement results are given in Fig. 12. The skin frictional stress without injection for each case was found to remain essentially unchanged, with the maximum case-to-case difference being 0.3% of the average value. Notably, the water injection case ${J}_{i}=0$ yielded a slight skin friction reduction, which corresponds to the water injection effect. Therefore, the baseline value for the dansyl-PEGMA solution injection may also be chosen as the skin friction ${C}_{f,0}=3.444\times {10}^{-3}$ for the ${J}_{i}=0$ case. In Table 2, the percentages of skin friction reduction $\left(1-{C}_{f}/{C}_{f,0}\right)\times 100$ (%) for three injection cases are listed. For the case ${J}_{i}=0.272\times {10}^{-6}g/\left({m}^{2}\cdot sec\right)$, the skin friction reduction was found to be 11.9%. Additionally, the skin friction reduction for the FDR-SPC case was found to be 9.49% (see Table 1). The similarity between the amounts of skin friction reduction by the two methods supports the presence of the Toms effect due to the present FDR-SPC.

## Discussions and conclusions

In the present study, the authors identified the skin frictional drag reducing mechanism of a novel polymer termed as FDR-SPC (Frictional Drag Reducing Self-Polishing Copolymer), which had been first synthesized by some of the present authors. The FDR-SPC was proposed as a revolutionary material to fulfil the polymer injection drag reduction by chemically releasing polyethylene glygol (PEG) into water. In spite of the previous reports of hydrodynamics performance for lab test and full-scale vessel test, the direct evidence of PEG release in water flows has not been confirmed. Here we present the results from a novel test method of in-situ planar laser-induced fluorescence (PLIF) method in which the concentration of the released fluorescein-labelled PEG can be quantified in terms of the intensity of the fluorescence in water flows. The PLIF measurement showed that the near-wall concentration of dansyl-PEG is observed to range from 1 to 2 ppm depending on the flow speed, which proves the existence of a drag reducing function for the FDR-SPC. In the concurrent measurement of skin friction, the present FDR-SPC specimen exhibited a skin friction reduction ratio of 9.49%. In the comparative experiment of mechanical polymer injection with similar mass flux, the skin friction was found to decrease by 11.9%. Therefore, the present results corroborate the presence of Toms effect from the FDR-SPC.

To the knowledge of authors, the FDR-SPC and associated FDR antifouling coating is the first practical implementation to accomplish the longstanding goal of turbulent skin friction reduction in a material basis. This can be paraphrased as the bulk flow control by means of surface chemistry. The present results can enlighten such capability in various academic fields other than marine hydrodynamics. For instance, this could inspire another novel material to improve hemodynamic performance in biomedical application. Consequently, the impact of the present study will not be merely limited to the reduction of greenhouse gas emission in marine transportation.

## Data Availability

The data that support the findings of this study are available from the corresponding author upon reasonable request.
